# Comparative Evaluation of Electrocoagulation for the Removal of Polyethylene and Polyvinyl Chloride Microplastics from Wastewater

**DOI:** 10.3390/polym18202501

**Published:** 2026-10-13

**Authors:** Bhavya Tiwari, Raj Kumar Arya, George D. Verros

**Affiliations:** 1 Department of Chemical Engineering, Dr. B. R. Ambedkar National Institute of Technology, Jalandhar 144008, Punjab, India; bhavyat.ch.24@nitj.ac.in; 2 Laboratory of Polymer and Dyes Chemistry and Technology, 54124 Thessaloniki, Greece; gdverros@gmail.com

**Keywords:** electrocoagulation, microplastic, polyethylene, polyvinyl chloride, sustainable wastewater treatment, sweep flocculation, electro-flotation

## Abstract

The persistence of microplastics in aquatic environments has created a need for effective methods of their removal from wastewater. The physicochemical differences among the polymers can influence their behaviour during wastewater treatment and that poses a polymer-specific removal challenge. This study comparatively evaluates the removal behaviour of polyethylene (PE) and polyvinyl chloride (PVC) microplastics from synthetic wastewater using aluminium–copper electrodes and investigates the effect of electrolysis time on treatment performance. Batch experiments were conducted in a 500 mL reactor at 3 V and 0.012 A for 30, 60, 90 and 120 min. Removal and sludge capture were evaluated gravimetrically, while FESEM–EDS was employed to characterize the recovered microplastics and identify elemental deposition on their surfaces. FESEM-EDS analysis revealed the deposition of aluminium-containing coagulation products on the recovered PE and PVC surfaces following electrocoagulation. Maximum floc removal was obtained at 60 min, reaching 49.72% sludge removal for PE and 27.18% for PVC, with corresponding sludge capture efficiencies of 65.21% and 63.14%, respectively. Electrolysis treatment times below and above 60 min produced unstable flocs that in turn reduced the removal efficiency. The specific energy consumption at the studied condition was ~0.07 kWh m^−3^ for both polymers. Overall, the results demonstrate polymer-dependent removal behaviour and highlight the influence of electrolysis time on the treatment performance of PE and PVC microplastics under investigated conditions.

## 1. Introduction

Plastic is one of the most widely used polymers in almost all sectors of human life, owing to its light weight, durability, moisture resistance, excellent heat and electrical insulation, low production cost and moldability. On the other hand, these specific properties are responsible for making plastic a recalcitrant pollutant that does not undergo complete weathering and degradation in nature. Global plastic production exceeds 430 million tons annually, with nearly 50% being single-use, out of which India’s annual contribution is approximately 9.3 million tons [1,2]. While plastic waste generation nearly doubled in the last decade, only about 9% is effectively being recycled. Every year, an estimated 8–52 million tons of plastic enter the world’s oceans. Land-based sources account for 80% of this pollution. Currently, there are 75–199 million tons of plastic waste circulating in marine ecosystems and India accounts for an estimated 12.9% of these global statistics [3]. This plastic, in the marine environment, undergoes gradual fragmentation through exposure to ultraviolet radiation, mechanical abrasion, thermal weathering and biological activities, producing progressively smaller particles known as microplastics (MPs). These MPs are defined as particles smaller than 5 mm in diameter and can originate from both primary sources (manufactured microbeads, industrial abrasives) and secondary sources arising from the fragmentation of larger plastic debris. In recent years, MPs have been increasingly detected in municipal wastewater, surface water, sediments and even drinking water systems. The most common types of plastic polymers used in our day-to-day life are polyethylene (low density and high density), polyethylene terephthalate (PET), polyvinyl chloride (PVC), polystyrene (PS), etc., which end up in wastewater treatment plants and ultimately reach natural water bodies. This raises significant environmental and public health concerns due to their presence, bioaccumulation potential and ability to absorb toxic pollutants such as heavy metals and organic contaminants [4].

Wastewater treatment plants (WWTPs) act both as sinks and secondary sources of microplastics entering aquatic ecosystems. Recent studies have reported microplastic presence in WWTPs’ effluents despite secondary and tertiary treatment stages [5,6,7]. While a substantial fraction of MPs are retained within sludge during treatment, smaller particles (<100 µm) escape into treated effluents [8]. As a result, there is a growing need for advanced and sustainable treatment technologies capable of effective targeting of these contaminants. Several technologies, including membrane filtration, dissolved air flotation, adsorption, magnetic separation, advanced oxidation processes and conventional coagulation–flocculation, have been investigated for MPs’ removal owing to their ability to separate particles with different sizes, densities and surface characteristics [9,10,11,12,13,14,15]. Although these techniques exhibit satisfactory performance and removal efficiencies under specific operational conditions, they often face challenges such as membrane fouling, high operational and maintenance cost, secondary sludge generation, reduced effectiveness over time and continuous chemical consumption [16]. Conventional coagulation–flocculation has shown partial removal [17], but efficiency strongly depends on particle size and polymer type. These limitations have stimulated increasing interest in developing sustainable, energy-efficient and chemical-minimized treatment that can effectively remove MPs from wastewater. Among these emerging technologies, electrocoagulation (EC) has gained considerable attention because it generates coagulant species in situ through the electrolytic dissolution of sacrificial aluminium electrodes. This mechanism eliminates the need for external chemical coagulants. The fundamental principles of EC are based on Faraday’s laws of electrolysis, established by Michael Faraday in 1834 [18]. These principles formed the basis for EC in which aluminium electrodes hydrolyse to generate Al^3+^ ions at the anode to form aluminium hydroxide flocs capable of destabilizing and entrapping suspended particles [19]. This technique has been extensively employed for the removal of suspended solids, heavy metals, dyes, oils, pharmaceuticals, nutrients and more recently, emerging pollutants such as MPs [20]. EC has shown promise for turbidity and colloidal removal, and limited experimental studies focus specifically on MPs without chemical coagulants [21].

Recent investigations of wastewater treatment plants have identified PE and PVC among the predominant polymers in wastewater influents and effluents [22]. PE and PVC are both widely used in plastic pipe materials in wastewater systems as they are both flexible, chemically inert and insulating in nature. However, over time, both these polymers undergo continuous mechanical wear, environmental degradation and physical friction. This persistent attrition causes microscopic polymer fragments and particulate debris to shed directly into wastewater streams, contributing to the growing burden of MPs in aquatic environments. In an advanced WWTP, PE accounted for 19.45% of the identified MPs, while PVC represented 3.90%, demonstrating their occurrence within treated wastewater systems [23]. Their presence is also evident in Indian wastewater systems. As reported by Parashar and Hait [24], low-density PE was predominantly found in sewage whereas PVC constituted the largest proportion of MPs recovered from sludge. Such differences in distribution indicate that polymer type may influence the partitioning and fate of microplastics during wastewater treatment. The relevance of PE and PVC is further associated with their contrasting physiochemical characteristics. PE has a relatively low density and is widely used in packaging, films, containers and consumer products, whereas PVC is a denser chlorine-containing polymer extensively used in pipes, construction materials, cable insulation and other industrial applications. Differences in polymer density and surface characteristics can influence whether MPs remain suspended, associated with flocs or become concentrated in sludge during treatment. For example, wastewater-treatment studies have reported that lower-density polymers such as PE are more likely to remain suspended, whereas denser polymers such as PVC can preferentially partition into settled sludge [25]. So, evaluating PE and PVC separately under identical electrocoagulation conditions provides an appropriate basis for examining polymer-dependent removal behaviour and determining whether differences in treatment performance are associated with the characteristics of the polymer itself.

Therefore, this study investigates the influence of electrolysis time on the removal performance of both polymer types under controlled operating conditions using an aluminium anode and copper cathode. The removal efficiency and sludge capture of PE and PVC are evaluated at different electrolysis durations to identify the suitable treatment time and compare the polymer-dependent removal behaviour. In addition, the untreated and residual microplastics are characterized using field-emission scanning electron microscopy (FESEM), (JEOL Ltd., Japan) and energy-dispersive X-ray spectroscopy (EDS) (Oxford Instruments, United Kingdom), to examine changes in particle morphology and surface elemental composition following electrocoagulation. The combined treatment-performance and characterization results are used to elucidate the differences in PE and PVC removal.

## 2. Mechanism of Microplastic Removal

The removal of MPs from wastewater involves a series of interconnected electrochemical and physicochemical processes that occur simultaneously as illustrated in Figure 1. When an electric current is applied, the sacrificial aluminium anode undergoes dissolution, releasing Al^3+^ ions into the solution which then undergo hydrolysis and form aluminium hydroxide species. These then aggregate into flocs which provide active sites for particle destabilization, adsorption and entrapment.

(1) Al → Al^3+^ + 3e^−^

While water reduction at the cathode produces hydrogen gas

(2) 2H_2_ O + 2e^−^ → H_2_ + 2OH^−^

The dissolved aluminium ions subsequently undergo hydrolysis to form various monomeric and polymeric hydroxide complexes before precipitating as amorphous aluminium hydroxide.

(3) Al^3+^ + 3H_2_O → Al(OH)_3_ + 3H^+^

These freshly formed aluminium hydroxide flocs provide reactive surfaces for the association and capture of suspended particles, facilitating particle aggregation through adsorption, bridging, and sweep flocculation [26]. Concurrently, hydrogen microbubbles produced at the cathode promote electro-flotation by attaching to the formed flocs and transporting them to the water surface. The denser aggregates settle under the effect of gravity. The overall removal efficiency is governed by the synergistic action of charge-mediated interaction, floc formation, adsorption and flotation. Other factors that affect the efficiency are current density, electrolysis time, pH, electrode material, electrolyte concentration and the physicochemical properties of MPs.

## 3. Materials and Methods

### 3.1. Materials

Polyvinyl chloride (PVC) powder (average molecular weight, Mw ≈ 43,000; Mn ≈ 22,000) was procured from Sigma-Aldrich (St. Louis, MO, USA) and polyethylene (PE) powder (purity > 99%) was purchased from Ultrananotech, India. Both were used as model MPs in the electrocoagulation experiments without further purification. Sodium chloride (NaCl, Extra Pure AR, 99.9%) was obtained from Sisco Research Laboratories (SRL), Mumbai, India, and was used as a supporting electrolyte to increase the electrical conductivity of the suspension. It also mimics the ionic strength of real wastewater. Tween 80 (Polyoxyethylene sorbitan monooleate; Product Code: T2610) was supplied by Rankem Laboratory Reagents, Avantor Performance Materials India Ltd., and employed as a non-ionic surfactant to facilitate homogeneous dispersion of hydrophobic MPs within the aqueous medium. All solutions were prepared using deionized (DI) water, and all chemicals were of analytical reagent (AR) grade and used as received without further purification.

The instruments employed in this study were an analytical weighing balance from RADWAG, Poland, supplied by LCGC Trucal and Services LLP, Hyderabad., a WANPTEK-CHAALAKAM SMPS Variable DC Power Supply 0–30 V & 0–05 A (150 W) GPS305D-02 Window 04 Digit Display, Kochi, Kerala, an SDFCL MS-300 SD Hot plate Magnetic Stirrer from Worli road Mumbai, Eisco Labs VITSZEE Chemical Fertilizers, Amazon.in, aluminium and copper electrode strips (100 × 19 mm), a Field Emission Scanning Electron Microscope, FESEM JEOL JSM-7610F-PLUS, and a Powder XRD (Bruker D8 Advance) from Billerica, MA, USA.

### 3.2. Preparation of Synthetic Wastewater

For each experiment, 0.10 g of PVC and PE powder were dispersed separately in 500 mL of deionized water to obtain an initial microplastic concentration of 200 mg L^−1^. To improve the dispersion stability of the hydrophobic particles, 1 mL of Tween 80 was added as a non-ionic surfactant. Subsequently, 0.5 g of sodium chloride (NaCl) was introduced into the suspension as a supporting electrolyte to increase the solution conductivity during treatment. The suspension was continuously stirred using a magnetic stirrer at approximately 300 rpm for 30 min to completely dissolve NaCl and promote uniform distribution of MP particles throughout the solution. The prepared suspension was then allowed to stand for 10–15 min before electrocoagulation for the solution to equilibrate. The synthetic wastewater was freshly prepared before each experimental run to ensure the consistency and reproducibility of the treatment process.

### 3.3. Electrocoagulation Setup

Electrocoagulation experiments were conducted separately for PE and PVC using 500 mL batches of the corresponding synthetic wastewater. These systems were operated under stable voltage of 3 V conditions using a regulated DC power supply with an average current of approximately 0.012 A. The electrolysis process was evaluated at four treatment durations of 30, 60, 90 and 120 min to investigate the effect of electrolysis time on MPs removal. Rectangular aluminium anodes and copper cathodes (5 × 2 × 0.2 cm each) were used with the inter-electrode gap of 2 cm throughout all experiments. The active area of electrodes corresponding to 10 cm^2^ was submerged into the EC chamber. The electrodes were connected to the DC power supply using insulated copper wires as illustrated in Figure 2. Prior to each experimental run, both electrodes were mechanically cleaned using fine emery paper/sandpaper to remove oxide layers and surface impurities, washed with dilute hydrochloric acid, rinsed with deionized water and air-dried.

During electrocoagulation, the suspension was continuously mixed using a magnetic stirrer at approximately 200 rpm to maintain homogeneous dispersion of PVC and PE particles. This stirring also promotes contact between the MPs and the generated coagulation products. The aluminium anode served as the sacrificial electrode for the generation of hydroxide flocs that capture the MPs. Upon completion of the electrolysis period, the electrical supply was switched off, and the suspension was allowed to settle under quiescent conditions to facilitate floc stabilization. The flocs were skimmed from the top onto a filter paper and the rest of the sludge-containing portion was filtered. Both the filter paper containing the retained solids from the top and the sludge were dried separately. The masses of the recovered filtrate and sludge were weighed using an analytical balance.

### 3.4. Removal Efficiency and Energy Consumption Calculation

The performance of the electrocoagulation process was evaluated in terms of microplastic removal efficiency and specific energy consumption. The top flocs were skimmed using a pre-weighed filter paper and dried to constant weight. The remaining suspension was passed through another pre-weighed filter paper and dried similarly (24 h at 35 °C). The increase in mass of the respective filter papers was used to determine the mass recovered in the floating flocs and sludge fractions. The percentage removal efficiency (η) was calculated based on the mass difference between the initial microplastic added to the electrocoagulation chamber and the residual microplastic remaining in the filtrate, according to:

(4) $$\eta \;\left(\%\right)=\;\frac{W_{r}}{W_{i}}\;\times \;100$$

where: *W_i_* = Initial mass of MPs added to the EC chamber (g).

*W_r_* = Mass of microplastics recovered from the floating fraction (g).

The sludge capture efficiency was evaluated by measuring the dry mass of the sludge collected after electrocoagulation and comparing it with the initial MPs mass. All calculations were performed using the average values obtained from experiments:

(5) $$\mathrm{Sludge}\;\mathrm{Capture}\;\mathrm{Efficiency}\;\left(\%\right)=\;\frac{W_{s}\;}{W_{i}}\times 100$$

where: *W_s_* = Dry mass of MPs recovered in the sludge fraction (g).

*W_i_* = Initial mass of MPs added (g).

Because the recovered solids may contain both microplastics and electrochemically generated coagulation products, blank electrocoagulation experiments were performed under the same operating conditions without the addition of microplastics. The dry mass recovered from these blank experiments was considered the background contribution from electrocoagulation-generated coagulation products and was used to correct the gravimetric measurements obtained from the microplastic-containing experiments. The blank-corrected masses were subsequently used for calculating the microplastic removal and sludge capture efficiencies. For each corresponding treatment condition, the blank-corrected mass of the recovered microplastic-associated fraction was calculated as:

(6) *M_corrected_* = *M_meas_* − *M_Blank_*

where: *M_corrected_* = Blank-corrected recovered mass (g).

*M_meas_* = Total dry mass measured in the corresponding fraction of MP-containing experiment (g).

*M_blank_* = Dry mass recovered from corresponding EC blank experiment without MPs (g).

The electrical energy consumed during electrocoagulation was calculated using:

(7) $$E=\;\frac{\left(V\times I\times t\right)}{V_{s}}$$

where: *E* = Energy consumption (JL^−1^).

*V* = Applied voltage (V).

*I* = Current (A).

*t* = Electrolysis time (s).

*V_s_* = Treated solution volume (L).

The energy consumption may also be expressed in kWhm^−3^ using the following relationship for comparison with the published literature [27,28,29]:

(8) $$E\;\left(\mathrm{kWh}m^{-3}\right)=\;\frac{\left(V\times I\times t\right)}{1000\times 3600\times V_{s}}$$

## 4. Results

### 4.1. Visual Observation

Visible changes were observed during electrocoagulation. Before treatment, the suspensions of PE and PVC both appeared uniformly turbid and milky white due to the presence of Tween 80 (Figure 3a). No sedimentation or aggregation was visible, indicating that the microplastics remained suspended. After the application of electric current, fine bubbles were observed to be evolving from the electrode surfaces which was indicative of hydrogen generation at the cathode and oxygen evolution at the anode, corresponding to previous electrocoagulation studies [30,31,32].

As the electrolysis proceeded, the suspension exhibited a gradual reduction in turbidity accompanied by the growth and settling of dense flocs. The flocs became more compact with increasing treatment time (Figure 3b). However, at longer treatment durations, changes in floc structure and distribution were observed, indicating that prolonged electrolysis did not necessarily result in progressively greater particulate capture. The PE and PVC treatments exhibited broadly similar overall treatment behaviour, characterized by floc formation and accumulation of particulate matter at the surface and within the settled solids. However, differences in the appearance and compactness of the recovered aggregates were also observed between the two polymers. PE-associated aggregates appeared comparatively more loosely structured, whereas PVC-associated aggregates showed relatively denser and more compact flocculated structures. These differences may be associated with the distinct physicochemical characteristics of the two polymers and their interaction with the electrochemically generated coagulation products.

The visual observations therefore indicate that both PE and PVC can become incorporated into the electrocoagulation-generated flocs, while the resulting aggregate characteristics may differ between the polymers. These observations are consistent with the quantitative removal results discussed in the subsequent sections and provide visual evidence of polymer-dependent treatment behaviour.

### 4.2. Removal Efficiency

The effect of electrolysis time on the removal of PE and PVC microplastics in the flocs is presented in Figure 4. The suitable treatment time for both the polymers was observed to be 60 min due to the highest removal efficiencies among the four treatment periods. At 30 min, the removal efficiencies were relatively low, reaching 15.69% for PE and 9.21% for PVC. The limited removal during the initial stage can be attributed to the insufficient dissolution of the aluminium anode. The inadequate amount of Al(OH)_3_ flocs required for effective charge neutralization and sweep flocculation are attributed to such low filtrate removal efficiencies for both polymers.

Increasing the electrolysis time to 60 min improved the removal efficiency, reaching 49.72% for PE and 27.18% for PVC. The significant improvement in the removal efficiency with the increase in electrolysis time is coherent with the results reported by various study groups previously [32,33]. In addition to sweep flocculation, electro-flotation resulting from hydrogen bubble evolution likely facilitates the transport of aggregates to the water surface contributing to microplastic separation [34].

A reduction in removal efficiency was observed after 90 min of treatment, likely associated with electrode passivation, excessive gas evolution, and partial fragmentation or restabilization of previously formed flocs. The numerical value for these reduced efficiencies was found to be 23.27% and 20.39% for PE and PVC, respectively. This decline after extended electrolysis may also be caused by saturation of adsorption sites on aluminium hydroxide flocs and disruption of the aggregated structures due to continuous agitation and bubble formation. The trend continues as the electrolysis time is further increased to 120 min. The removal efficiency decreases even lower, reportedly 7.05% in PE and 13.81% in PVC. Similar effects of treatment times on the removal efficiency have been reported in other electrocoagulation studies treating suspended particles and microplastics [35,36].

The two polymers exhibited varying removal efficiency throughout the experiments, with PE being higher than PVC at the current selected conditions. The difference may be linked with variation in particle density, surface properties and interactions with electrochemically generated flocs. The lower density of PE may favour its flotation with gas bubbles and the surface chemistry can influence the interaction with aluminium hydroxide. In contrast, PVC possesses a higher density and different surface characteristics, reducing its transportation with the flocs even under identical conditions. Similar polymer-dependent behaviours have been reported in recent investigations, highlighting the influence of polymer type and its chemical properties on electrocoagulation performance [37].

### 4.3. Sludge Capture Efficiency

The sludge capture efficiencies of PE and PVC microplastics at different electrolysis times are presented in Figure 5. At 30 min, the sludge capture efficiencies were 28.93% for PE and 40.36% for PVC, which indicates limited incorporation of MPs into the settled or aggregated flocs. With the increase in the electrolysis time to 60 min, enhanced efficiencies were reported to reach 65.21% for PE and 63.14% for PVC. The higher recovery can be due to the increased dissolution of the aluminium anode and the formation of abundant Al(OH)_3_ flocs. The larger flocs captured the polymers and were easy to remove from the top whereas the remaining portion was filtered as sludge. However, extending the electrolysis time to 90 min resulted in a substantial decline in sludge capture efficiency to 19.44% for PE and 14.43% for PVC. A further increase in electrolysis time to 120 min resulted in PE and PVC particles being settled at the bottom of the EC chamber without sufficient floc formation and being removed as sludge without any incorporation into the hydroxide matrix. The respective efficiencies reported at this time were 44.44% for PE and 45.52 for PVC but these should not be treated as increases in removal efficiency as there were no flocs due to floc destabilisation and fragmentation of formed coagulants. These trends need to be further verified by carrying out current and voltage optimization at varying time intervals [38].

### 4.4. Energy Consumption

The specific energy consumption of the electrocoagulation process was evaluated to assess the electrical input associated with the treatment using Equations (7) and (8). The calculated consumption was found to be 259.2 J/L or 0.072 kWh m^−3^ for PVC and 265.68 J/L or 0.0738 kWh m^−3^ for PE. The relatively low electrical requirement indicates that the electrocoagulation treatment achieved microplastic separation with a comparatively low energy input under the conditions examined.

The energy requirement is influenced by the electrical operating conditions and treatment duration, as well as the extent of aluminium dissolution and floc formation during electrocoagulation. Therefore, the observed energy consumption should be interpreted together with the corresponding microplastic removal performance rather than as an independent measure of treatment effectiveness. The energy savings relative to previously reported values in the literature were calculated. Compared with the energy consumption of 0.78 kWhm^−3^ reported by Perren, Wojtasik and Cai [27], the present study achieved energy savings of 90.77% for PVC and 85.60% for PE. Similarly, relative to the lower end of the conventional EC energy range (0.5 kWhm^−3^) as reported in the literature [28,39,40], the energy savings were 90.54% for PVC and 85.24% for PE. Subair, K, Chellappan, Rahuman, Hridya, Devi, Salkka, Indu, Pugazhendhi and Chinglenthoiba [29] also demonstrated removal efficiencies exceeding 90% for polystyrene microbeads under optimized current densities accompanied by increased energy demand.

The results suggest that increasing the applied current or voltage could potentially improve microplastic removal by increasing the rate of aluminium dissolution and in turn enhancing coagulant formation. Such improvements, however, would inevitably increase operational costs and electrode consumption. This leads to the consideration during optimization of electrical parameters that not only removal efficiency but also the trade-off between treatment performance, energy consumption, and process economics are relevant in order to evaluate the complete process and declare it as efficient and suitable on a large scale.

### 4.5. Field Emission Scanning Electron Microscopy (FESEM) and Energy Dispersive X-Ray Spectroscopy (EDS) Analysis

Field emission scanning electron microscopy coupled with energy dispersive X-ray spectroscopy was employed to investigate the surface morphology and elemental composition of PE and PVC microplastics during electrocoagulation, with particular emphasis on the deposition of electrochemically generated coagulation products. Representative FESEM micrographs were obtained for the untreated microplastics, residual particles recovered from the floating flocs, and sludge generated after electrocoagulation. Simultaneously, EDS analysis was performed to determine the elemental composition of the corresponding samples and to assess the presence of electrochemically generated metal hydroxides in the floc structure.

#### 4.5.1. Untreated Microplastics Before EC

FESEM images (Figure 6) of the untreated PE and PVC microplastics revealed the original morphology of the polymer particles prior to electrocoagulation. The PE particles exhibited irregular flake-like morphology with relatively smooth surfaces, less compacted and flattened in some regions, whereas PVC images show more uniform granular particles, nearly spherical grains, and rough and cauliflower-like surfaces, denser and more compacted than PE. The absence of agglomeration, surface deposits, or secondary phases indicates that the virgin microplastics remained physically stable before treatment. The particle size was analysed using ImageJ 1.54g, Wayne Rasband and contributors National Institute of Health, USA and in a range from 22.665 µm to 25.399 µm in the case of PE and 157.415 µm to 161.130 µm in the case of PVC, while the surface appeared free from inorganic impurities or corrosion products.

The smooth and chemically inert surface of the untreated polymers justify their high colloidal stability in aqueous suspension, making spontaneous sedimentation difficult under normal conditions. Such behaviour is characteristic of microplastics due to their hydrophobic surfaces and relatively low surface charge, which inhibits natural aggregation.

EDS spectra of the untreated particles (Figure 7) confirmed the elemental composition of the polymers. PE predominantly exhibited a strong carbon (C) signal with minimal oxygen (O) contribution. In contrast, PVC showed carbon as a major element (71.63%) with a distinct chlorine (Cl) contribution of 27.68%. This chlorine abundance is consistent with the reported chlorine-containing polymer structure of PVC [41]. Importantly, no detectable aluminium (Al) signals were observed, indicating the absence of detectable AL-containing product in the analysed region.

#### 4.5.2. Analysis of Microplastics Recovered from Floating Flocs

FESEM images (Figure 8) demonstrated that both PE and PVC underwent morphological changes during the EC treatment. The sample for analysis was obtained after the 60 min treatment cycle for both the polymers. Compared with the untreated polymers, both polymers exhibited surface deposition and agglomeration, indicating their attachment to the generated coagulant species but with significant differences in appearance. PE particles largely retained their original morphology, although portions of their surfaces were covered by deposited material and some particles appeared interconnected through localized bridges. In contrast, PVC particles formed denser agglomerates with extensive surface coverage that formed compact structures, and the individual PVC particles were not distinguishable like they were before treatment. These observations are indicative of the incorporation of PE and PVC into the floating floc fraction with more aggregation in PVC as seen under the investigated conditions.

The EDS spectra of the top floating flocs in Figure 9 indicate substantial changes in surface elemental composition following treatment. Carbon remained the predominant element in both PE and PVC samples even after the treatment, reflecting the intact polymeric backbone of the MPs. Compared with untreated polymers, both PE and PVC present increased oxygen and aluminium contents after EC with PE showing 24.92 wt% of oxygen and 5.23 wt% aluminium, while PVC showed 12.48 wt% O and 1.63% Al. The enrichment of oxygen and aluminium on both polymers confirms the deposition of aluminium-based hydroxide flocs generated during the treatment process. Only a trace amount of chlorine (0.29 wt%) was detected on the treated PVC sample, whereas no measurable chlorine signal was reported for the PE spectrum. The absence of a significant chlorine peak in the PVC spectrum likely reflects the localized nature of EDS analysis and the fact that the analysed surface was predominantly covered by deposited floc material rather than exposed PVC substrate. Since EDS is a surface-sensitive, point-based technique, elemental composition depends on the specific region selected for analysis and may not represent the bulk polymer composition. The combined FESEM and EDS analyses demonstrate that both PE and PVC interacted with the generated coagulation products successfully.

#### 4.5.3. Analysis of Electrocoagulation Sludge

The sludge generated during EC exhibited a heterogeneous three-dimensional floc structure composed of metal hydroxides, embedded MPs, and aggregated coagulation products as shown in Figure 10. Unlike the relatively isolated particles observed in the untreated samples, the recovered sludge consisted of large interconnected aggregates with irregular and porous morphologies. FESEM images show that numerous MP particles were either partially covered or embedded within the coagulation matrix. In several regions, individual polymer particles were difficult to distinguish because they were fully covered by amorphous metal hydroxide precipitates. The flocs exhibited interconnected pores and cavities, forming an irregular three-dimensional network. The formation of large interconnected aggregates is consistent with the increased sludge capture observed experimentally, as microplastic particles associated with the flocs could be recovered within the settled sludge fraction.

At higher magnification, the polymer surfaces appeared coated with fine granular precipitates. These small deposits correspond to electrochemically generated Al(OH)_3_ species produced through anodic dissolution and subsequent hydrolysis as detected by EDS analysis. The observed association of these deposits to the polymer supports the incorporation of MPs within the coagulation matrix.

EDS spectra in Figure 11 further characterized the elemental composition of recovered sludge. The sludge from both the samples exhibited prominent carbon signals originating from the presence of MPs, oxygen associated with metal hydroxides, and Al from the electrode material used. PVC sludge additionally showed detectable chlorine signals, consistent with the presence of PVC-derived material in the recovered sludge. The concurrent detection of carbon, oxygen, and aluminium in the sludge supports the association of the microplastic particles with the aluminium-containing coagulation matrix. These observations, together with the FESEM morphology, indicate that the microplastics were incorporated into the floc structure during electrocoagulation.

## 5. Discussions

### 5.1. Comparison Between PE and PVC

The FESEM-EDS observations provide evidence of the association of aluminium-containing coagulation products with the recovered PE and PVC MP surfaces. The recovered particles were observed within or partially covered by the coagulation matrix, while the EDS spectra showed increased oxygen and aluminium signals compared with the untreated polymer surfaces. These observations indicate the deposition of aluminium-based coagulation products on the microplastic surfaces during electrocoagulation. The differences observed between PE- and PVC-associated aggregates may reflect differences in particle characteristics and their interaction with the generated coagulation products; however, the present FESEM observations do not provide sufficient evidence to conclude that the polymer morphology itself underwent substantial structural alteration during treatment. Therefore, the observed changes are interpreted primarily as surface coating, deposition, and incorporation of microplastic particles into the coagulation matrix rather than morphological degradation of the polymers. However, these factors alone are not responsible for explaining every related mechanism. Factors like particle size, distribution, polymer hydrophobicity, surface chemistry and other molecular-level interactions are required to be explored to get the complete picture [42]. The lower density and hydrophobic nature of PE may favour its association with hydrogen bubbles and flotation, while differences in surface chemistry may influence its interaction with aluminium-containing coagulation products [43]. However, the denser morphology observed for PVC did not translate into higher overall removal efficiency, as PE achieved greater removal under the investigated conditions.

The presence of aluminium-containing material on both PE and PVC supports their incorporation into the electrocoagulation-generated floc phase. The formation of larger aggregates and the partial coverage of recovered microplastic particles are consistent with physical entrapment and particle–floc association during treatment. These observations complement the quantitative removal results by providing microscopic evidence that aluminium-based coagulation products were associated with the recovered microplastic fraction. Similar incorporation of MPs into the EC flocs has been reported with flocculation, charge neutralization, adsorption and physical entrapment identified as important removal processes [44]. The limited morphological changes observed in the recovered particles suggest that, under the conditions investigated in the present study, removal occurred primarily through phase separation and floc incorporation rather than extensive morphological degradation [45].

### 5.2. Comparison with the Literature

To compare the observed differences of the current electrocoagulation process with the previously published studies on microplastic removal, Table 1 summarizes them. Such comparisons are useful for understanding how polymer type and operating conditions influence the treatment process and performance. Direct comparison, though possible, is limited due to differences in particle size, electrode material, configuration, current density, reactor design, wastewater composition, ambient conditions, etc.

The PE removal efficiency observed in this study is consistent with the high removal reported for PE microbeads in the study by Perren et al. (2018) [27] to some extent. Their study reported the effectiveness of aluminium-based EC for microbead removal with efficiencies exceeding 90%. That study also highlighted the importance of operating conditions in achieving efficient treatment. The agreement between their findings and the present work supports the suitability of aluminium-based electrocoagulation for hydrophobic polyethylene particles, while differences in particle characteristics and reactor conditions may account for variations in performance.

Similar findings have been reported by Subair, K, Chellappan, Rahuman, Hridya, Devi, Salkka, Indu, Pugazhendhi and Chinglenthoiba [29], where the EC was carried out for polystyrene (PS) removal using different aluminium and stainless-steel electrode configurations. Their investigation identified removal efficiencies approaching 99% depending on electrical loading, electrolyte concentration, treatment time, electrode configurations and other varying factors. These results support the observation in the present study that treatment performance depends not only on the polymer being treated but also on the conditions under which EC is operated.

The detailed investigation by Mateo et al. [46] is particularly relevant to the behaviour of polymers at extended electrolysis time. That study further showed that EC performance depends on the applied electrical charge which adversely affects MPs removal beyond the optimum level. Using micro polystyrene particles, the authors demonstrated that excessive electrical charge may restabilize previously formed flocs, thereby reducing removal efficiency despite prolonged treatment. This observation is consistent with the decline observed after optimum treatment performance. Their work also showed that aluminium electrodes consistently outperformed iron electrodes because of the superior adsorption characteristics of aluminium hydroxide flocs.

The difference between PE and PVC observed in the present study is also coherent with the previous study by Gabisa and Ratanatamskul [37] who examined the simultaneous removal of PET, PP, and PS using graphene-assisted electrocoagulation. It was reported that polymer characteristics, along with electrolyte concentration, applied voltage and other operating variables, affected removal behaviour. The present study similarly suggests that differences in particle properties and their interactions with the generated coagulation products can contribute to polymer-specific removal behaviour [48].

Another study by Sezer, Topkaya, Aksan, Veli and Arslan [47] optimized electrocoagulation for treating real biological wastewater treatment plant effluent containing mixed microplastics and achieved approximately 99% removal using Taguchi optimization. Unlike the present work, which investigated single-polymer synthetic wastewater to isolate polymer-specific behaviour, their study focused on process optimization in a complex wastewater matrix. The comparison indicates that although higher removal efficiencies can be achieved under optimized conditions and in different reactor configurations, the present process demonstrates excellent performance while maintaining remarkably low energy consumption and simple operating conditions.

Overall, comparison with previous studies confirms that electrocoagulation consistently achieves removal efficiencies exceeding 90% for polyethylene and polystyrene microplastics under optimized conditions, whereas polymer properties and operating parameters strongly influence treatment performance. One of the principal contributions of the present study is the direct comparison between PE and PVC under identical electrocoagulation conditions, providing evidence that polymer composition plays a significant role in both filtrate removal and sludge capture efficiency. Furthermore, the exceptionally low specific energy consumption (~0.07 kWh m^−3^) demonstrates that effective microplastic removal can be achieved under low-current operation, improving the economic feasibility of electrocoagulation for wastewater treatment.

## 6. Conclusions

This study demonstrated the potential of electrocoagulation for the removal of polyethylene (PE) and polyvinyl chloride (PVC) microplastics from synthetic wastewater using aluminium electrodes. Electrolysis time influenced the removal performance of both polymers, with 60 min identified as the suitable electrolysis time under investigated operating conditions. Under these conditions, maximum floc removal efficiencies of 49.72% for PE and 27.18% for PVC were achieved, accompanied by sludge capture efficiencies of 65.21% and 63.14%, respectively. The higher removal observed for PE compared with PVC may be associated with differences in polymer characteristics. However, the difference in particle size between the two polymers may limit attribution of the observed behaviour to polymer chemistry.

FESEM–EDS observations supported a removal mechanism involving charge-mediated interactions, adsorption, sweep flocculation, physical entrapment, and electro-flotation, with aluminium-containing coagulation products deposited on the recovered microplastic surfaces. The reduction in removal and sludge capture at longer electrolysis durations may be associated with electrode passivation, prolonged gas evolution, and floc destabilization or restabilization.

The specific energy consumption at 60 min was 0.0738 kWh m^−3^ for PE and 0.0720 kWh m^−3^ for PVC, corresponding to 265.68 and 259.2 J L^−1^, respectively. Compared with the reported energy consumption of 0.78 kWh m^−3^, these values represent reductions in energy consumption of 90.54% for PE and 90.77% for PVC. Relative to 0.5 kWh m^−3^, the corresponding reductions were 85.24% and 85.60%, respectively. These results indicate that the investigated electrocoagulation system achieved microplastic removal under relatively low electrical energy input.

A direct comparison of PE and PVC under identical electrocoagulation conditions demonstrates polymer-dependent removal behaviour and highlights the influence of electrolysis time and microplastic characteristics on treatment performance. The findings provide a basis for further investigation of operating parameters, including voltage and electrolysis time, and for evaluation of the process using mixed polymers and real wastewater systems.

## Figures and Tables

**Figure 1:**
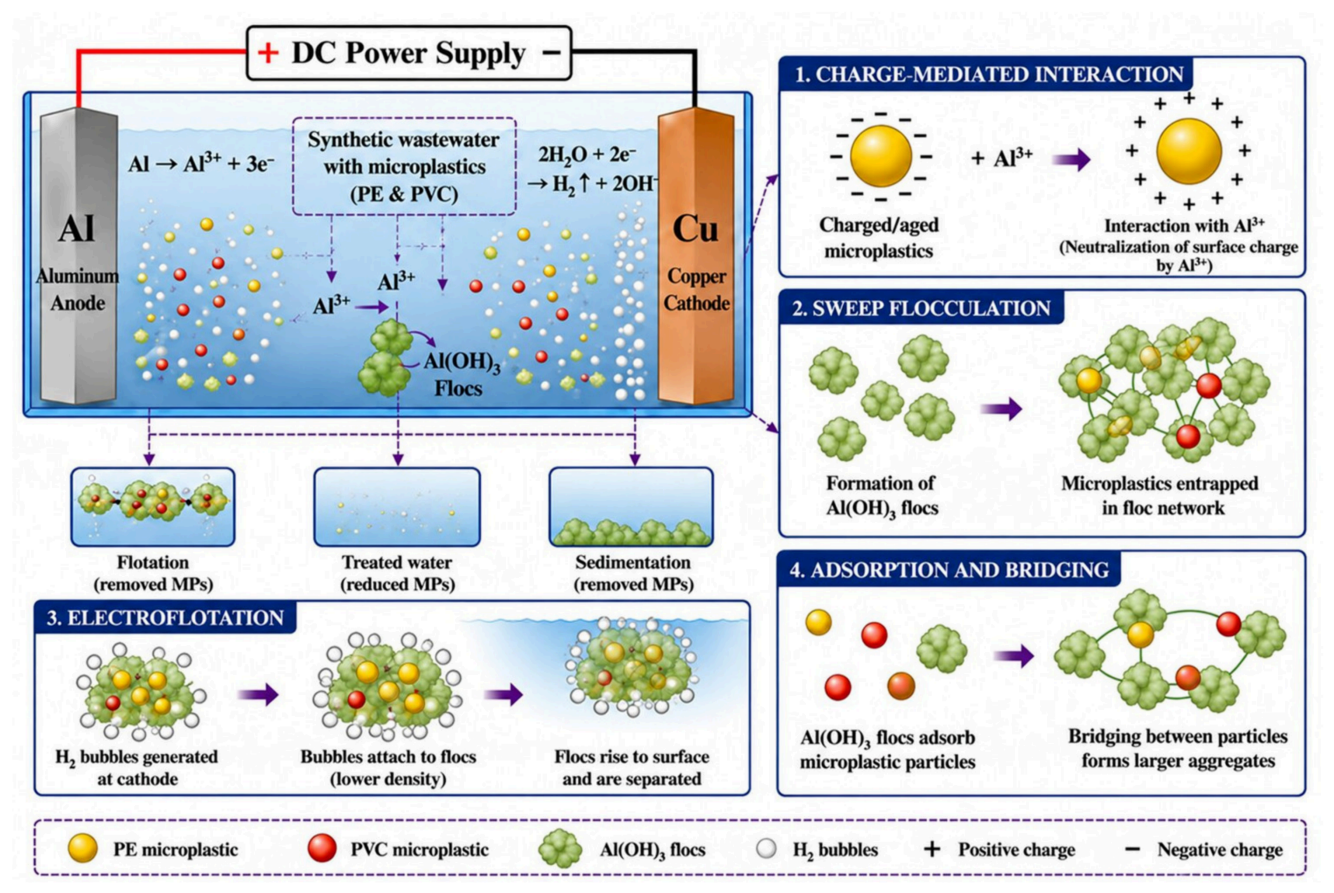
Schematic representation of potential mechanism involved in microplastic (PE and PVC) removal during electrocoagulation (EC).

**Figure 2:**
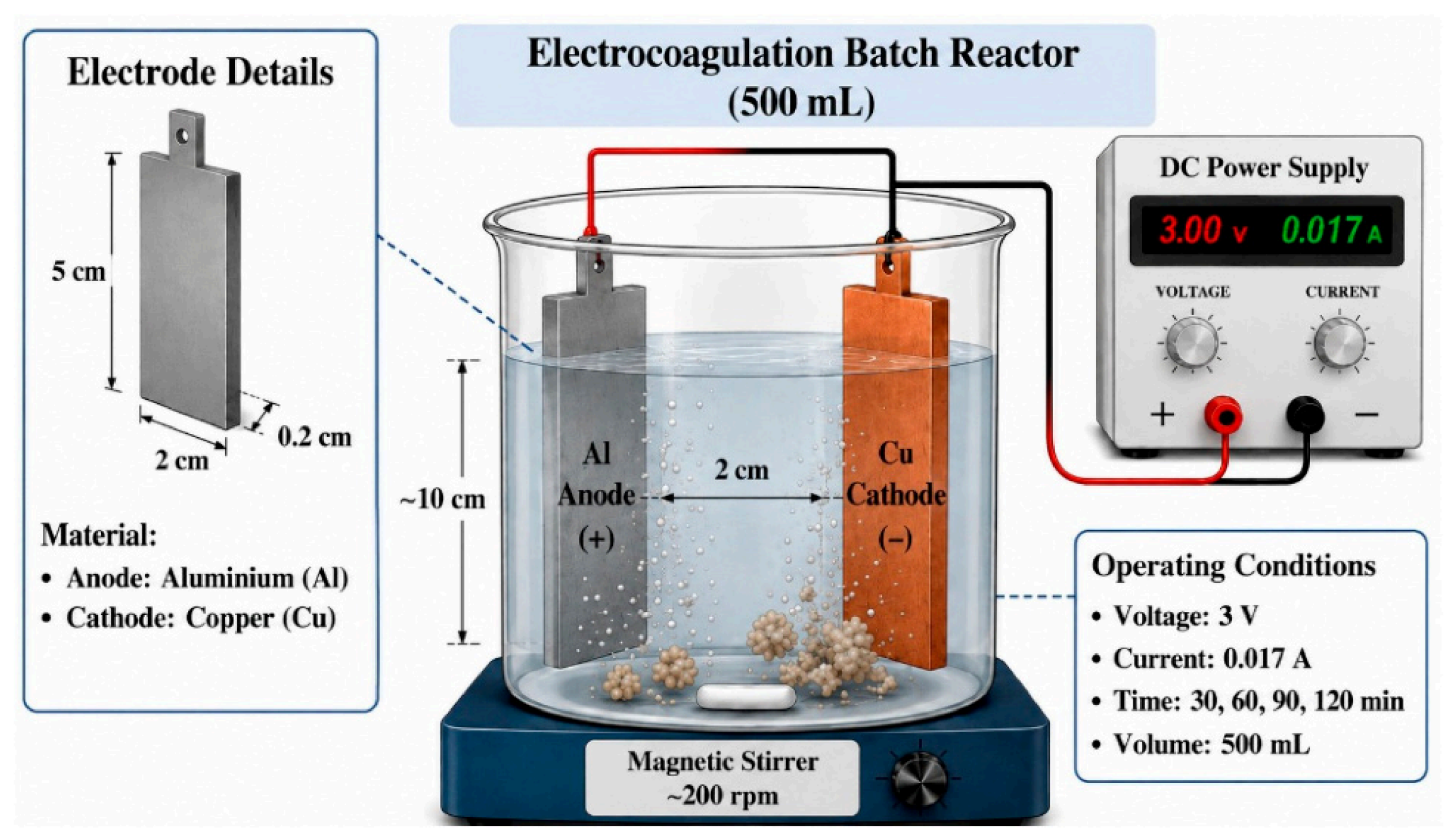
Laboratory setup of electrocoagulation.

**Figure 3:**
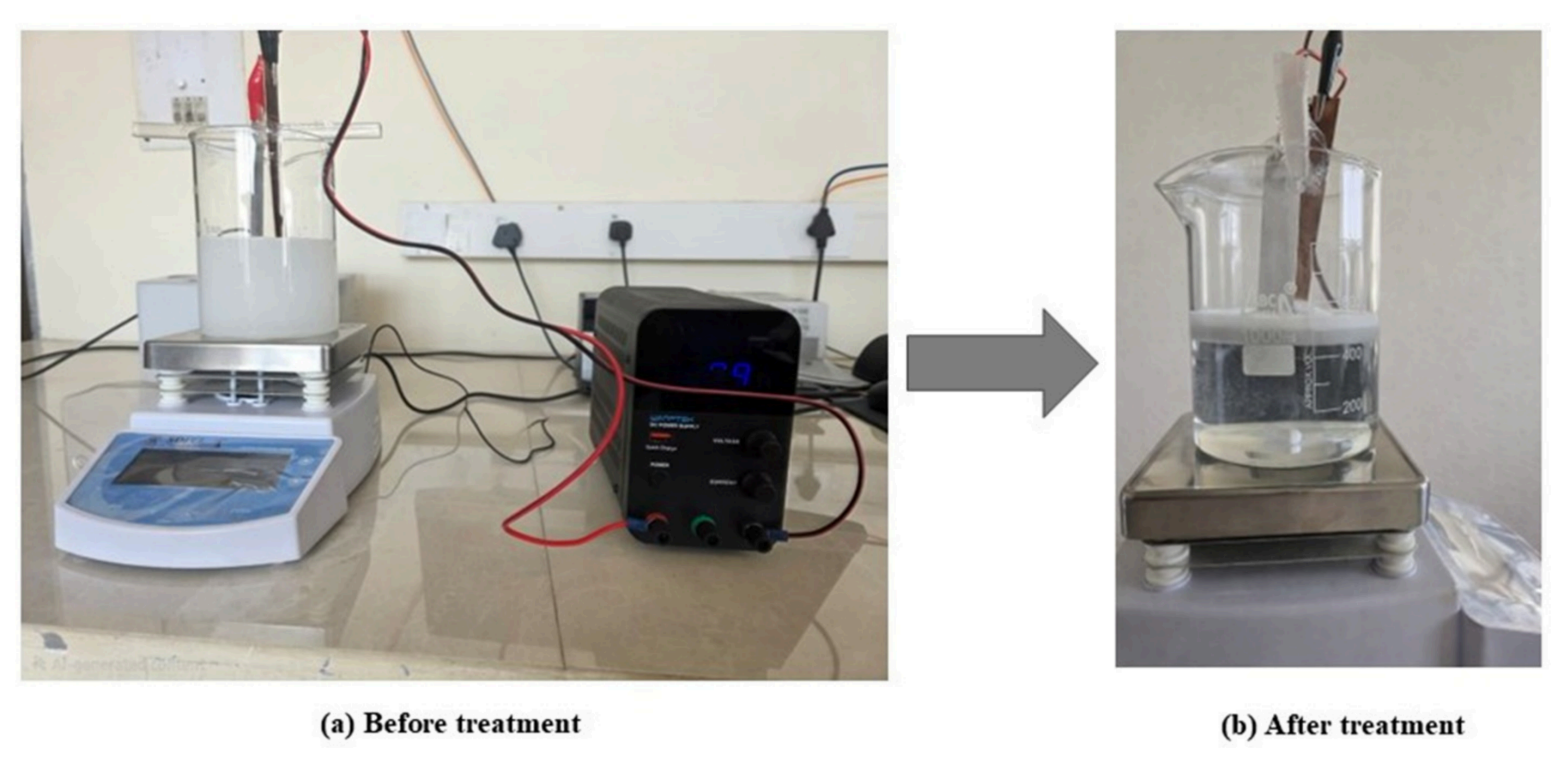
Visual appearance of synthetic MPs wastewater (**a**) before electrocoagulation showing a uniformly turbid suspension and (**b**) after electrocoagulation showing clarified supernatant with settled flocs.

**Figure 4:**
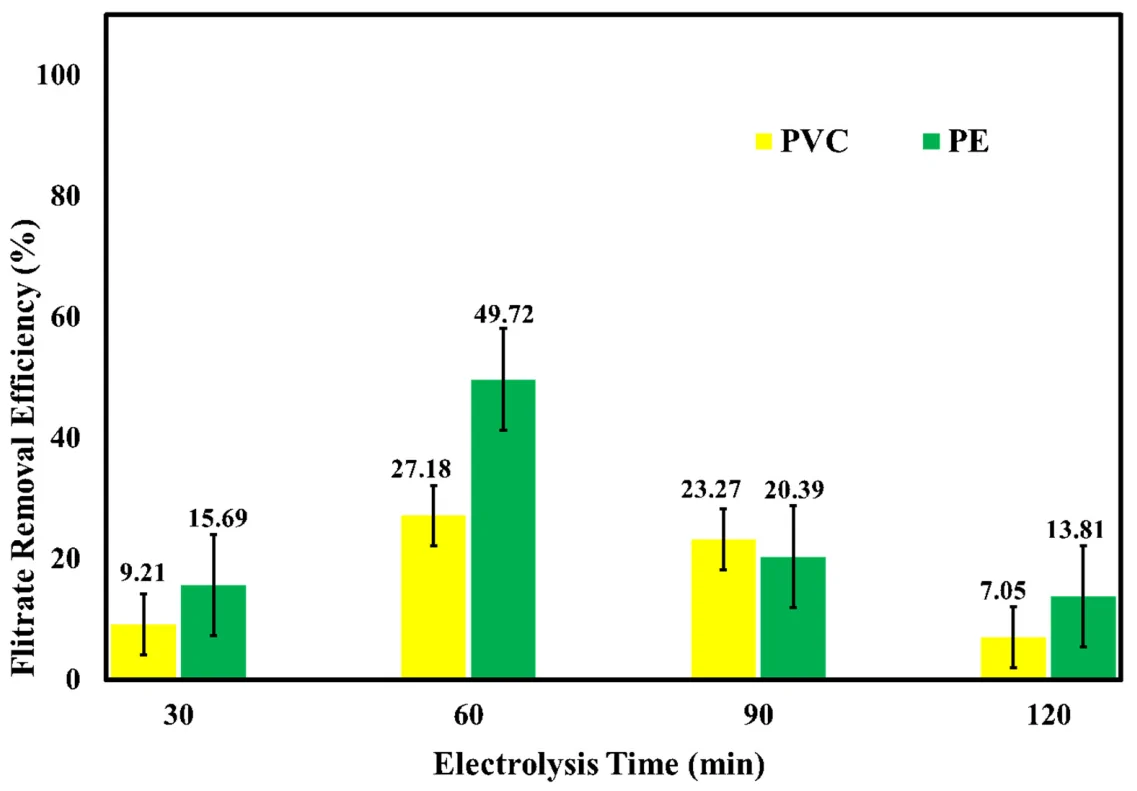
Comparative filtrate removal efficiencies of polyethylene (PE) and polyvinyl chloride (PVC) during electrocoagulation at different electrolysis times.

**Figure 5:**
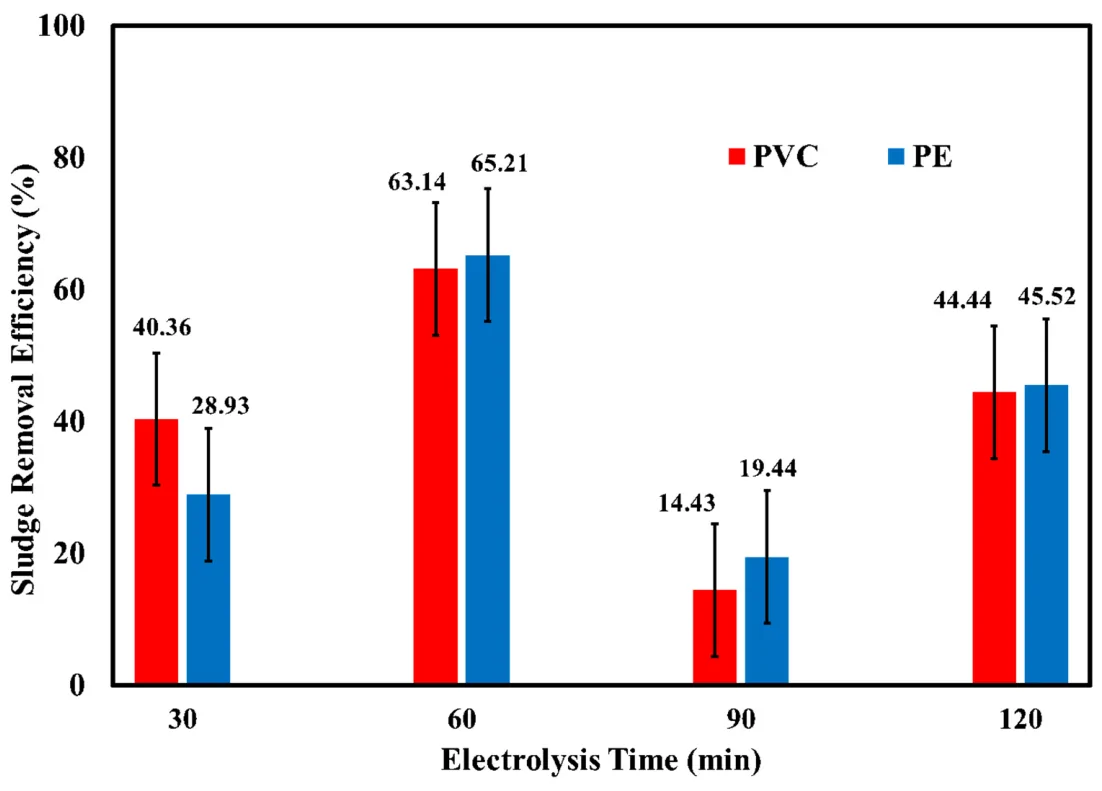
Comparative sludge capture efficiencies of polyethylene (PE) and polyvinyl chloride (PVC) during electrocoagulation at different electrolysis times.

**Figure 6:**
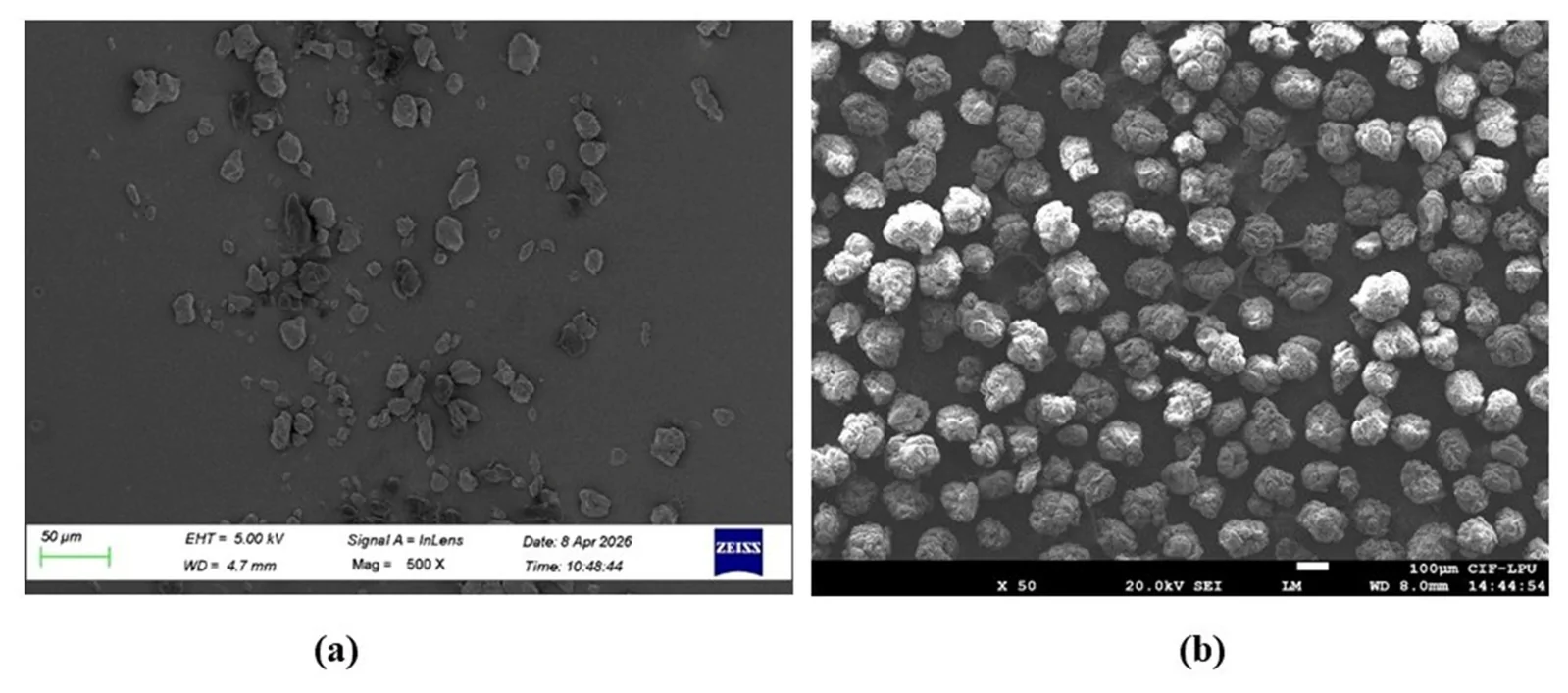
FESEM micrographs of untreated commercial (**a**) PE and (**b**) PVC microplastics.

**Figure 7:**
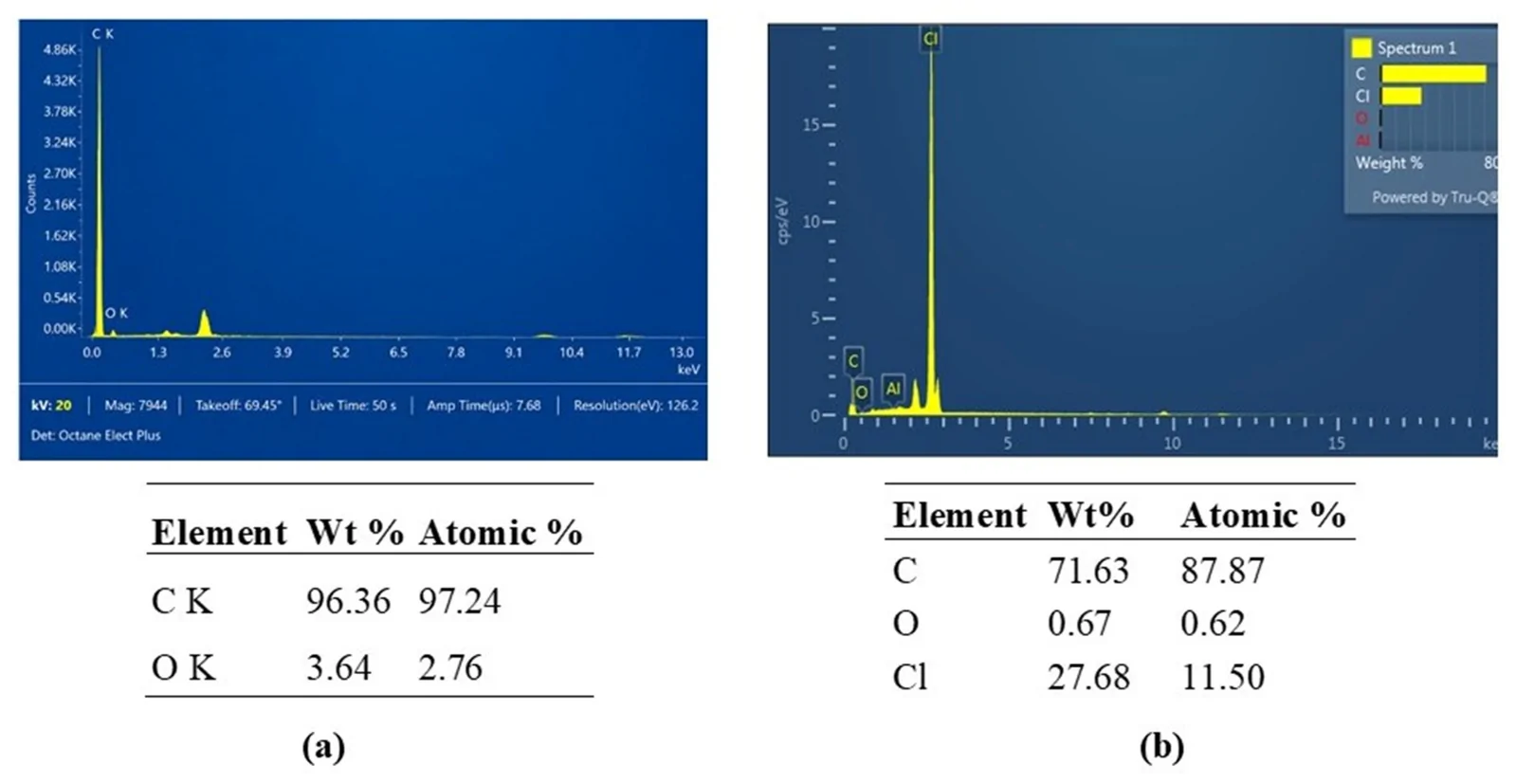
EDS spectra of untreated commercial (**a**) PE and (**b**) PVC microplastics.

**Figure 8:**
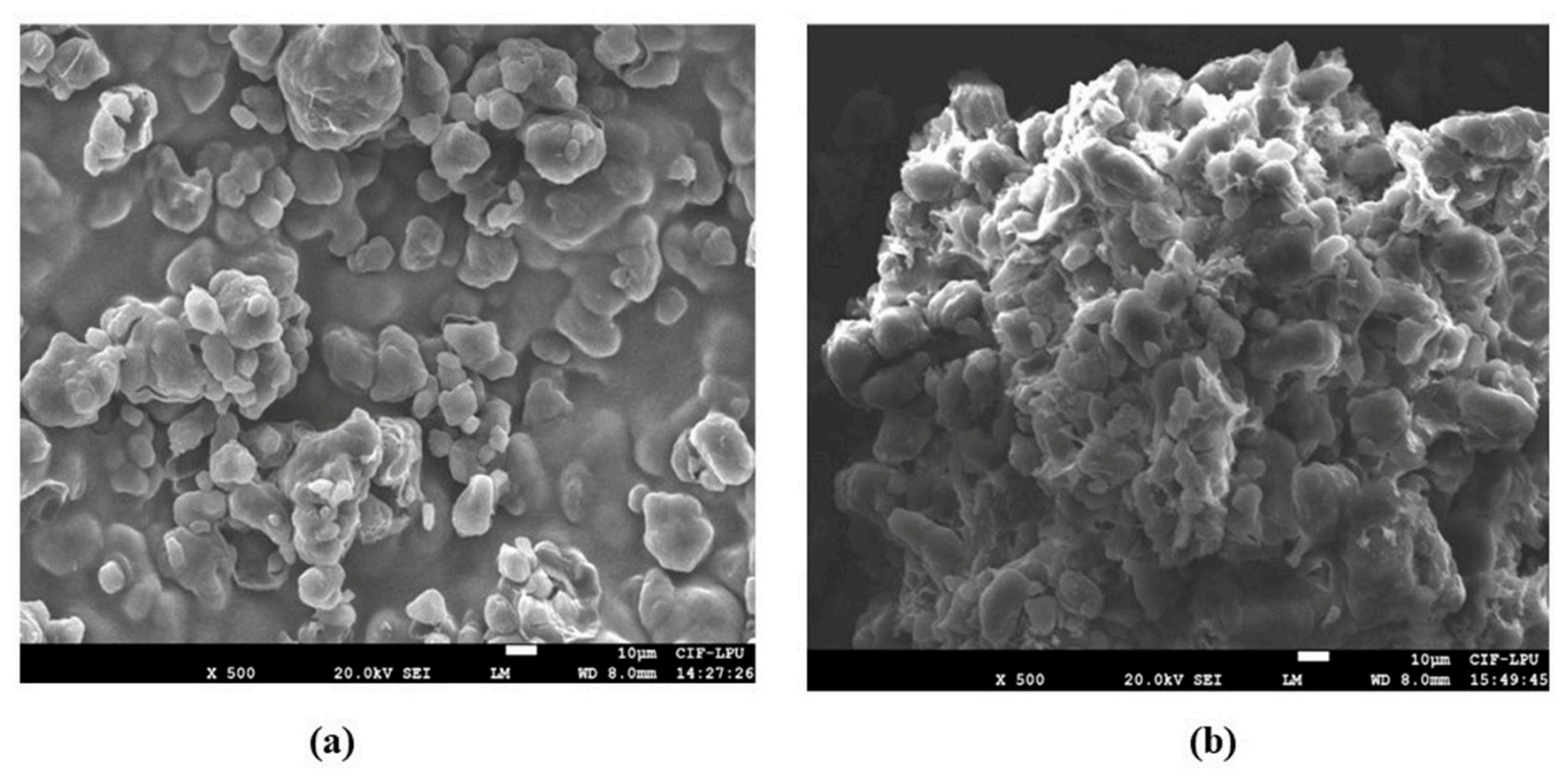
FESEM micrographs of (**a**) PE and (**b**) PVC filtrate after EC.

**Figure 9:**
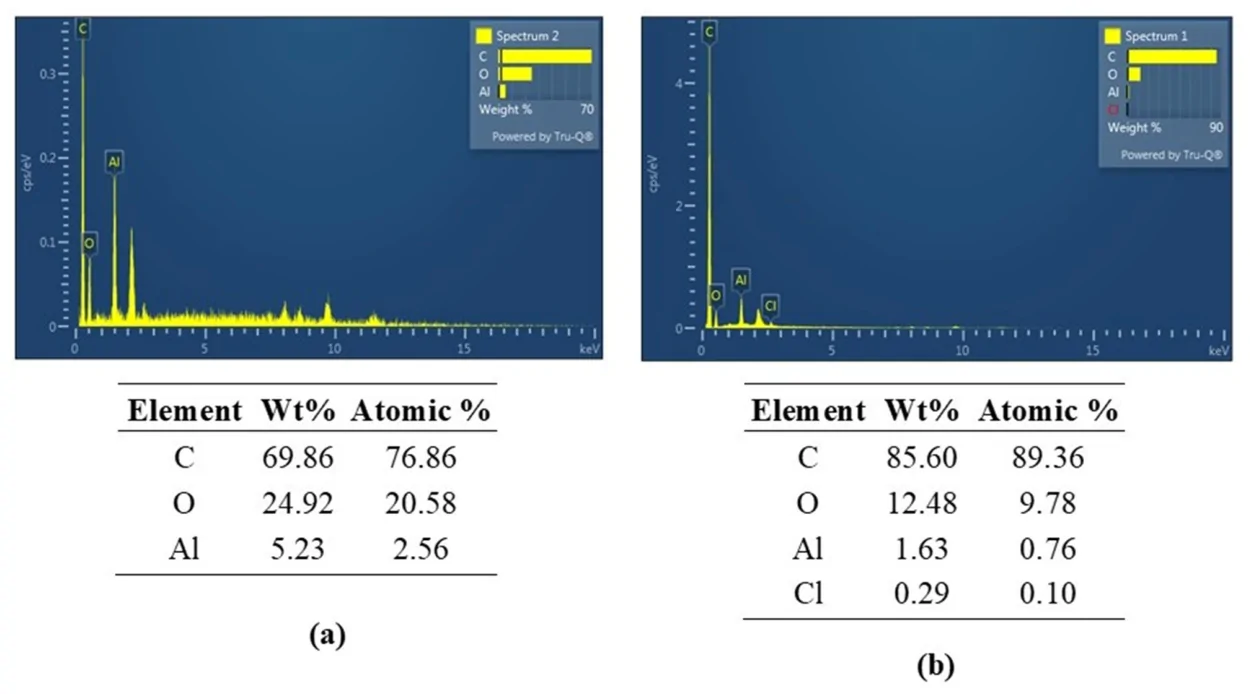
EDS spectra of (**a**) PE and (**b**) PVC filtrate after EC.

**Figure 10:**
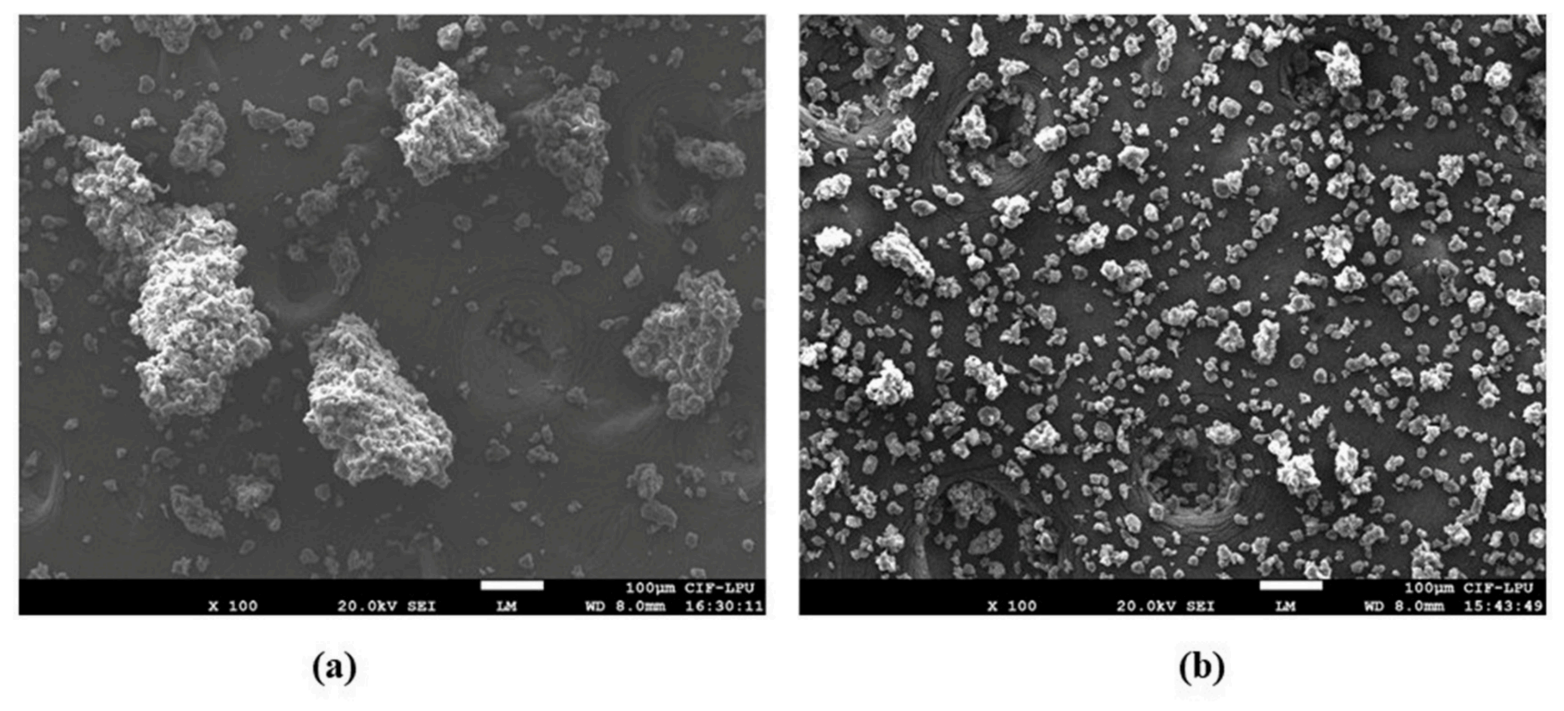
FESEM micrographs of (**a**) PE and (**b**) PVC sludge after EC.

**Figure 11:**
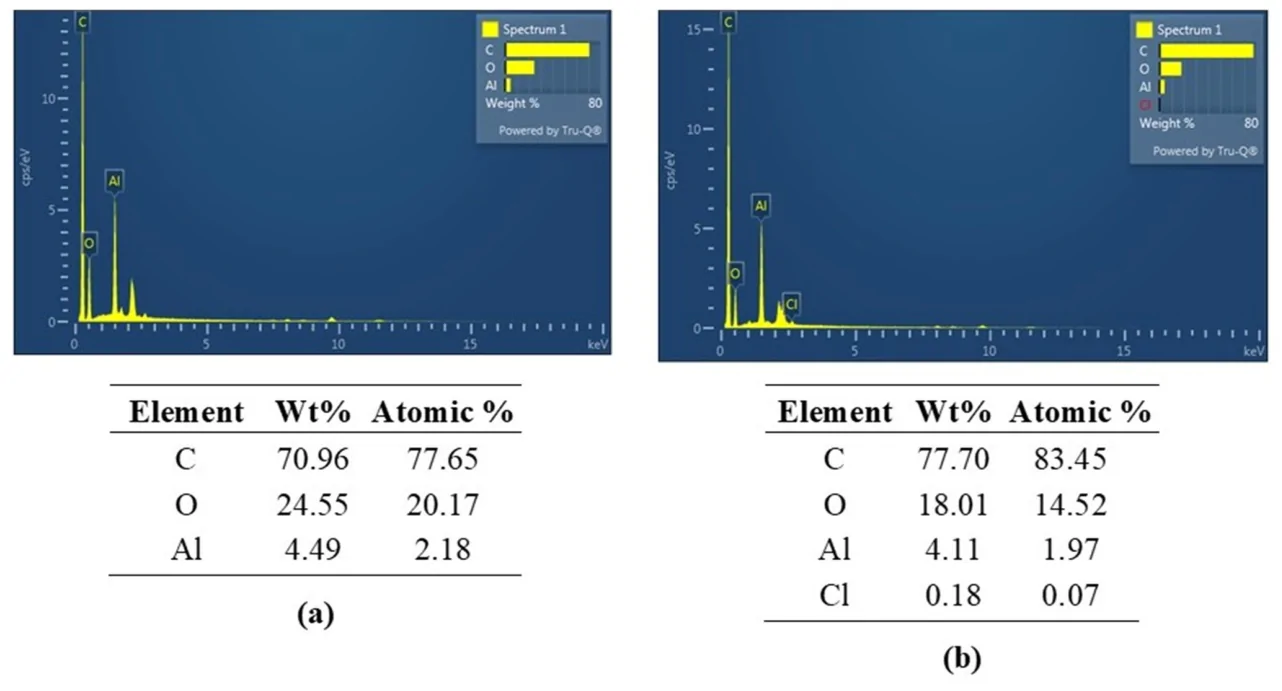
EDS spectra of (**a**) PE and (**b**) PVC sludge after EC.

**Table 1:** Comparison of current study with the existing literature.

Study	Polymer	Electrode Material	Reactor/Operating Conditions	Maximum Removal (%)	Energy Consumption
Present study	PE	Al	3 V, 0.012 A, 60 min	65.21%	0.072 kWh m^−3^
Present study	PVC	Al	3 V, 0.012 A, 60 min	63.14%	0.0738 kWh m^−3^
Perren, Wojtasik and Cai [27]	PE microbeads	Al	pH 7.5; NaCl 2 g L^−1^; current density 11 A m^−2^; 60 min; bipolar parallel configuration	99.24%	~£0.05 m^−3^ (operating cost)
Subair, K, Chellappan, Rahuman, Hridya, Devi, Salkka, Indu, Pugazhendhi and Chinglenthoiba [29]	PS	Al–Al, Al–SS, SS–SS	0.2 A; pH 7; KCl 0.5 g L^−1^; 50 min; cylindrical hollow wire-mesh Al electrodes; monopolar parallel configuration	~99%	Not directly comparable
Mateo, Zhang, Piedra, Ruiz, Miranda and Rodríguez [46]	Micro polystyrene	Al and Fe	16.3 A m^−2^; 30 min; 0.0075 M Na_2_SO_4_ 0.5 g L^−1^ μPS; 50–100 µm; 30 mm gap 2.00 L	>90%	0.13 kWh m^−3^
Gabisa and Ratanatamskul [37]	PET, PP, PS	Al/graphene	10 V for Al/graphene, pH 5.5, and 0.1 M NaCl	High (>95%)	Not directly comparable
Sezer, Topkaya, Aksan, Veli and Arslan [47]	Mixed MPs	Al	pH 9, 1.905 mA/cm^2^ and 15 min (Taguchi optimization)	~99%	0.049$ m^−3^ (operating cost)

## Data Availability

The original contributions presented in this study are included in the article. Further inquiries can be directed to the corresponding author.
